# Proton Magnetic Resonance Spectroscopy Lactate/N-Acetylaspartate Within 48 h Predicts Cell Death Following Varied Neuroprotective Interventions in a Piglet Model of Hypoxia–Ischemia With and Without Inflammation-Sensitization

**DOI:** 10.3389/fneur.2020.00883

**Published:** 2020-09-04

**Authors:** Raymand Pang, Kathryn A. Martinello, Christopher Meehan, Adnan Avdic-Belltheus, Ingran Lingam, Magda Sokolska, Tatenda Mutshiya, Alan Bainbridge, Xavier Golay, Nicola J. Robertson

**Affiliations:** ^1^Department of Neonatology, Institute for Women's Health, University College London, London, United Kingdom; ^2^Medical Physics and Engineering, University College London NHS Foundation Trust, London, United Kingdom; ^3^Department of Brain Repair and Rehabilitation, Institute of Neurology, University College London, London, United Kingdom

**Keywords:** neonatal encephalopathy, magnetic resonance spectroscopy, hypoxia–ischemia, piglet, therapeutic hypothermia, neuroprotection

## Abstract

Despite therapeutic hypothermia, survivors of neonatal encephalopathy have high rates of adverse outcome. Early surrogate outcome measures are needed to speed up the translation of neuroprotection trials. Thalamic lactate (Lac)/N-acetylaspartate (NAA) peak area ratio acquired with proton (^1^H) magnetic resonance spectroscopy (MRS) accurately predicts 2-year neurodevelopmental outcome. We assessed the relationship between MR biomarkers acquired at 24–48 h following injury with cell death and neuroinflammation in a piglet model following various neuroprotective interventions. Sixty-seven piglets with hypoxia–ischemia, hypoxia alone, or lipopolysaccharide (LPS) sensitization were included, and neuroprotective interventions were therapeutic hypothermia, melatonin, and magnesium. MRS and diffusion-weighted imaging (DWI) were acquired at 24 and 48 h. At 48 h, experiments were terminated, and immunohistochemistry was assessed. There was a correlation between Lac/NAA and overall cell death [terminal deoxynucleotidyl transferase dUTP nick end labeling (TUNEL)] [mean Lac/NAA basal ganglia and thalamus (BGT) voxel *r* = 0.722, white matter (WM) voxel *r* = 0.784, *p* < 0.01] and microglial activation [ionized calcium-binding adapter molecule 1 (Iba1)] (BGT *r* = −0.786, WM *r* = −0.632, *p* < 0.01). Correlation with marker of caspase-dependent apoptosis [cleaved caspase 3 (CC3)] was lower (BGT *r* = −0.636, WM *r* = −0.495, *p* < 0.01). Relation between DWI and TUNEL was less robust (mean diffusivity BGT *r* = −0.615, fractional anisotropy BGT *r* = 0.523). Overall, Lac/NAA correlated best with cell death and microglial activation. These data align with clinical studies demonstrating Lac/NAA superiority as an outcome predictor in neonatal encephalopathy (NE) and support its use in preclinical and clinical neuroprotection studies.

## Introduction

Neonatal encephalopathy (NE) secondary to intrapartum hypoxia–ischemia is a significant cause of brain injury in term infants affecting 2–3 per 1,000 live births in the UK (1). Therapeutic hypothermia (HT) has reduced mortality and disability in survivors of NE [relative risk (RR) 0.75, 95% CI 0.68–0.83, number needed to treat (NNT) = 7] (2). However, despite treatment, there remains a 24–30% mortality rate and 22–44% risk of moderate to severe disability at 18 months following moderate to severe NE (3, 4). NE has a complex and multifactorial etiology; however, over the last decade, preclinical (5) and clinical (6) studies suggest that coexisting infection and inflammation with hypoxia–ischemia (HI) exacerbate brain injury. A strong association exists between fetal infection/inflammation (e.g., chorioamnionitis, funisitis), perinatal brain damage, and neurodisability (7).

In single (8) and multicenter (9) studies of NE babies who have been cooled, the ^1^H magnetic resonance spectroscopy (MRS) thalamic lactate (Lac)/N-acetylaspartate (NAA) peak area ratio acquired within 15 days of birth accurately predicts neurodevelopmental outcomes. Refinements in the spectral fitting including threonine (Thr) and N-acetylaspartylglutamate (NAAG) in the fitting function can improve the analysis of the spectrum in the regions close to Lac and NAA, respectively, and better signal to noise at 3 Tesla (3T) have optimized the predictive accuracy of Lac/NAA (8). Using a threshold of 0.39, the sensitivity and specificity of BGT Lac/NAA for 2-year motor outcome was 100% and 97%, cognition 90% and 97% and language 81% and 97%, respectively (8). In the TOBY Xenon early-phase clinical neuroprotection trial, adverse outcomes were correctly identified in 95.65% of cases by basal ganglia and thalamus (BGT) Lac/NAA, whereas prediction of adverse outcome using fractional anisotropy (FA) was 78.79% (10). Using Lac/NAA peak area ratio as a qualified biomarker in the clinical context in a small proof-of-concept neuroprotection trial therefore avoids substantial financial and opportunity costs associated with large randomized controlled trials (RCTs).

Over the last two decades, we have used BGT and white matter (WM) Lac/NAA as one of our primary outcome markers in neuroprotection studies of adjunct therapies with HT in our piglet model (11–15). The piglet model allows for regional assessment of brain immunohistochemistry at 48 h with analyses including quantification of terminal deoxynucleotidyl transferase dUTP nick end labeling (TUNEL)-positive cells, assessment of neuroinflammation [ionized calcium-binding adapter molecule 1 (Iba1) ramification index], and quantification of cleaved caspase 3 (CC3), a marker of caspase-dependent apoptosis.

Given the importance of MRI biomarkers in neonatal clinical neuroprotection trials and the translational pathway from preclinical to clinical RCTs, our aim was to assess: (i) the relationship between MR biomarkers [^1^H MRS metabolite ratios, mean diffusivity (MD), FA], acquired at 24 and 48 h following injury, and brain cell death and neuroinflammation at 48 h in the piglet following various neuroprotective interventions; (ii) brain immunohistochemistry differences related to the Lac/NAA peak area ratio clinical threshold of 0.39 (this ratio accurately predicts 2-year motor, cognitive, and language outcomes in babies with NE) (8). In this study, we included retrospective data from different injuries (hypoxia–ischemia, hypoxia, inflammation-sensitization) and neuroprotective interventions (HT alone and with magnesium or melatonin) to assess the relation between MR biomarkers and immunohistochemistry in the piglet model.

## Methods

### Animal Experiments, Surgical Preparation, and Intensive Care Management

All animal experiments were approved by the UCL Ethics Committee and performed according to UK Home Office Guidelines [Animals (Scientific Procedures) Act, 1986]. The study complies with Animal Research: Reporting of *in vivo* Experiments (ARRIVE) guidelines.

Piglets were anesthetized and surgically prepared as described previously (11, 13, 14, 16, 17). In brief, all piglets were sedated with intramuscular midazolam and anesthetized with inhaled 3–4% v/v isoflurane. A tracheostomy was performed, and piglets were intubated (Smiths Medical, Ashford, Kent, UK) and ventilated (SLE 2000 Infant Ventilator, Surrey UK) for the duration of the experiment. Carotid vascular occluders (OC2A, *in vivo* Metric, Healdsburg, CA, USA) were sited for all studies, except study 3. Umbilical venous and arterial access were obtained (arterial catheter Vygon 2.5Fr, venous catheter−2Fr double lumen), and a peripherally inserted central venous catheter (Vygon 2Fr Nutriline) was sited in the proximal forelimb for infusion of intravenous drugs. Piglets were transferred onto a specialized incubator following surgery where continuous vital signs, multichannel electroencephalography (EEG) (Nicolet EEG, Natus), and cerebral near-infrared spectroscopy (NIRS) were monitored. Sedation was maintained with infusion of fentanyl (4 μg/kg/h) and inhaled isoflurane.

Piglets were cared for in accordance with local neonatal intensive care guidelines throughout the experiment. Following insult, maintenance fluid was restricted to 40 ml/kg/day. Ventilation settings were titrated according to arterial blood gas measurements. Mean arterial blood pressure (MABP) was maintained >35 mmHg using infusions of dopamine, dobutamine, noradrenaline, and adrenaline as required. Electrolytes, urea and creatinine, and blood glucose were monitored. All piglets received benzylpenicillin and gentamicin. 10% calcium gluconate (0.5 ml/kg) and salbutamol (4 μg/kg) were used to treat hyperkalemia. Seizures were treated with intravenous phenobarbitone followed by phenytoin if persistent.

### Study Selection and Variations in Study Design

**Figure 1:**
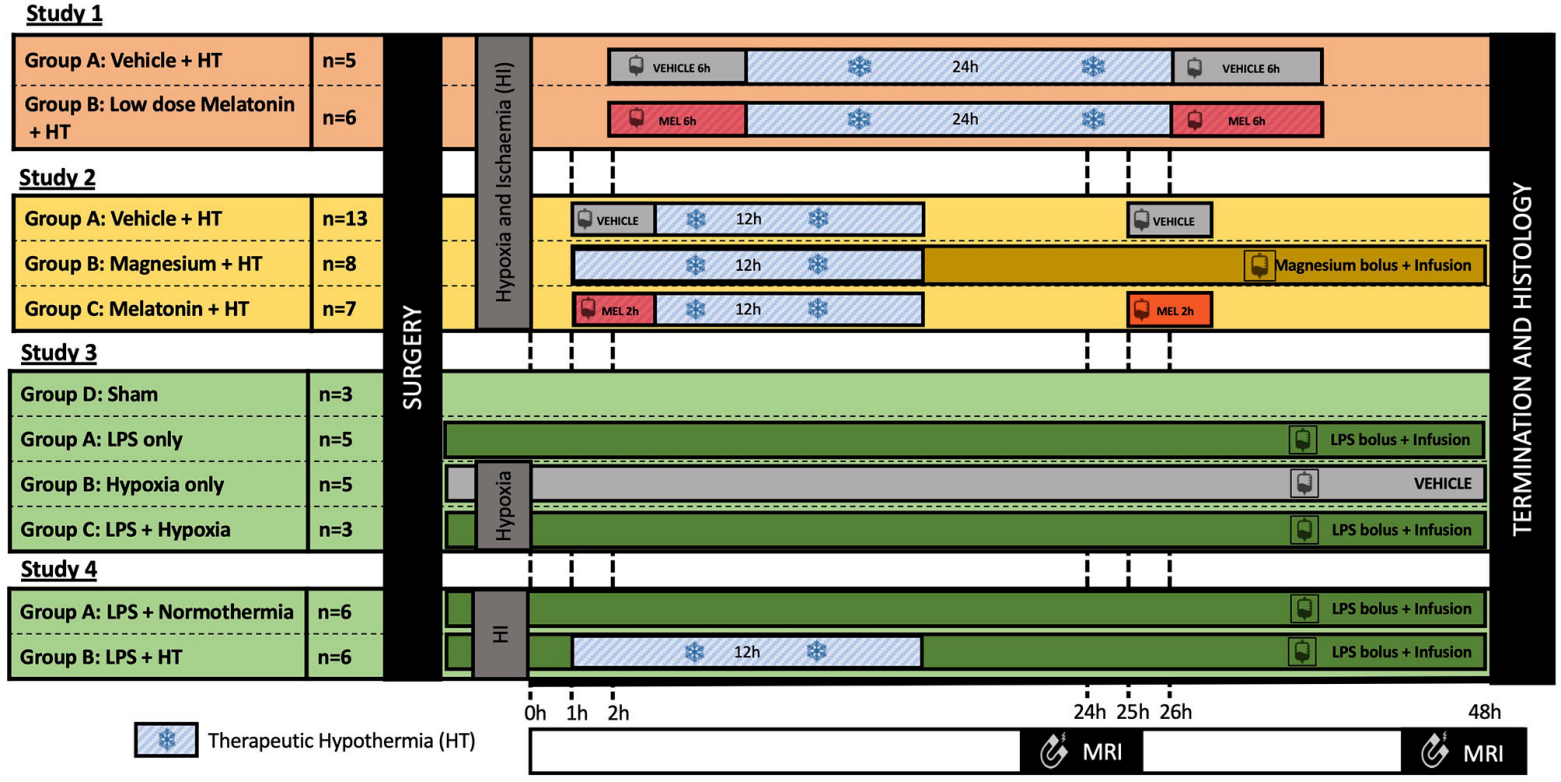
Summary of the experimental protocols. All experiments were 48 h in length; however, cerebral insults and neuroprotective interventions varied between studies. In all studies, 3T–^1^H magnetic resonance spectroscopy (MRS) was acquired at 24 and 48 h after insult, and immunohistochemistry was assessed at 48 h. Study 1 (11): Piglets were subjected to a transient hypoxia–ischemia (HI) followed by therapeutic hypothermia (HT) for 24 h, started at 2 h after insult. Piglets were randomized to either: (i) vehicle infusion or (ii) melatonin infusion over 6 h at 2 h after HI, repeated at 26 h. Study 2 (13, 17): Piglets were subjected to HI insult followed by cooling for 12 h started 1 h after insult. Piglets were randomized to: (i) vehicle infusion; (ii) magnesium bolus 1 h post insult followed by continuous infusion over 48 h; or (iii) melatonin infused over 2 h at 1 h and 25 h after insult. Study 3 (14): Piglets were randomized to: (i) control (saline bolus and infusion), (ii) lipopolysaccharide (LPS) (bolus followed by a continuous infusion); (iii) hypoxia; or (iv) LPS and hypoxia 4 h after bolus. Study 4: All piglets were subjected to LPS bolus 4 h prior to HI and continuous infusion over 52 h. Animals were then randomized to either normothermia or 12 h HT from 1 to 13 h after insult.

This study was a retrospective, secondary analysis of four preclinical neuroprotection piglet studies (11, 13, 14, 17). The study protocols evolved, reflecting optimization and development of study designs over the years, and are shown in Figure 1. For full details of the study methodology and results, please refer to publications (11, 13, 14, 17).

All studies lasted 48 h; however, studies varied according to brain injury protocols, duration of HT, and neuroprotective agents used. Primary outcome measures for all studies were identical; MRS was acquired at 24 and 48 h after insult, and immunohistochemistry was assessed at 48 h using the same methodology. Acquisition using the clinical 3T scanner (Philips Achieva) was introduced during study 1 to enhance the translational relevance of our preclinical model. Prior to this, ^1^H MRS was acquired using a 9.4T MRI scanner. Only piglets with MRS data at 3T and immunohistochemistry data were included in this secondary analysis. Piglets scanned at 9.4T or with no 3T MRS data were excluded.

#### Brain Injury

Piglets in studies 1 (11) and 2 (13, 17) were subjected to HI. Carotid artery occluders were inflated to induce brain ischemia and the fraction of inspired oxygen (FiO_2_) was reduced to 4% and titrated according to response. The HI insult for study 1 was conducted within the bore of a 9.4T MRI. During HI, the ^31^P MRS β-NTP peak height was continuously monitored, and the FiO_2_ was titrated to keep the β-NTP peak height between 30 and 40% of its original height for a period of 12.5 min. The insults for studies 2–4 were conducted outside the MRI. For these studies, insult duration and FiO_2_ titration were determined by MABP (target between 26 and 30 mmHg), duration of flat EEG, arterial blood gas measurements (target lactate between 10 and 12), and NIRS oxidized-cytochrome C levels. Persistent severe hypotension (MABP < 25 mmHg) or bradycardia was an indication to terminate the insult.

Piglets in studies 3 (14) and 4 underwent inflammation-sensitization with *Escherichia coli* liposaccharide (LPS) (Sigma O55:B5) prior to cerebral injury. A bolus of 2 μg/kg LPS followed by an infusion 1 μg/kg/h for the duration of the experiment was given. At 4 h after infusion, piglets in study 3 were subjected to a hypoxia-only insult by reducing FiO_2_ to 4%. In study 4, piglets were subjected to an HI insult as described in studies 1 and 2.

#### Neuroprotective Interventions

All piglets in studies 1 and 2 were cooled to 33.5°C using a servo-controlled water mattress (Tecotherm); however, protocols varied between the studies. In study 1, piglets were cooled from 2 h after HI over a duration of 24 h. Piglets in study 2 were cooled from 1 h after HI for a total duration for 12 h. No piglets in study 3 received HT. In study 4, piglets in the HT treatment arm were cooled for 12 h. All piglets that received HT were rewarmed at a controlled rate of 0.5°C/h to the target temperature of 38°C. Normothermia at 38.5°C was maintained by the water mattress.

Various neuroprotective agents were used in these studies. Piglets in study 1 received either an intravenous melatonin infusion at 2 and 26 h after HI at 5 mg/kg over 6 h or vehicle at the same volume and infusion rate. In study 2, piglets received (i) magnesium as a loading bolus of 180 mg/kg followed by continuous infusion 8 mg/kg/h at 1 h after HI; (ii) melatonin at 18 mg/kg over 2 h at 1 h and 25 h after HI; or (iii) vehicle at the same volume and rate. No additional agents were used in studies 3 or 4.

### Magnetic Resonance Imaging

**Figure 2:**
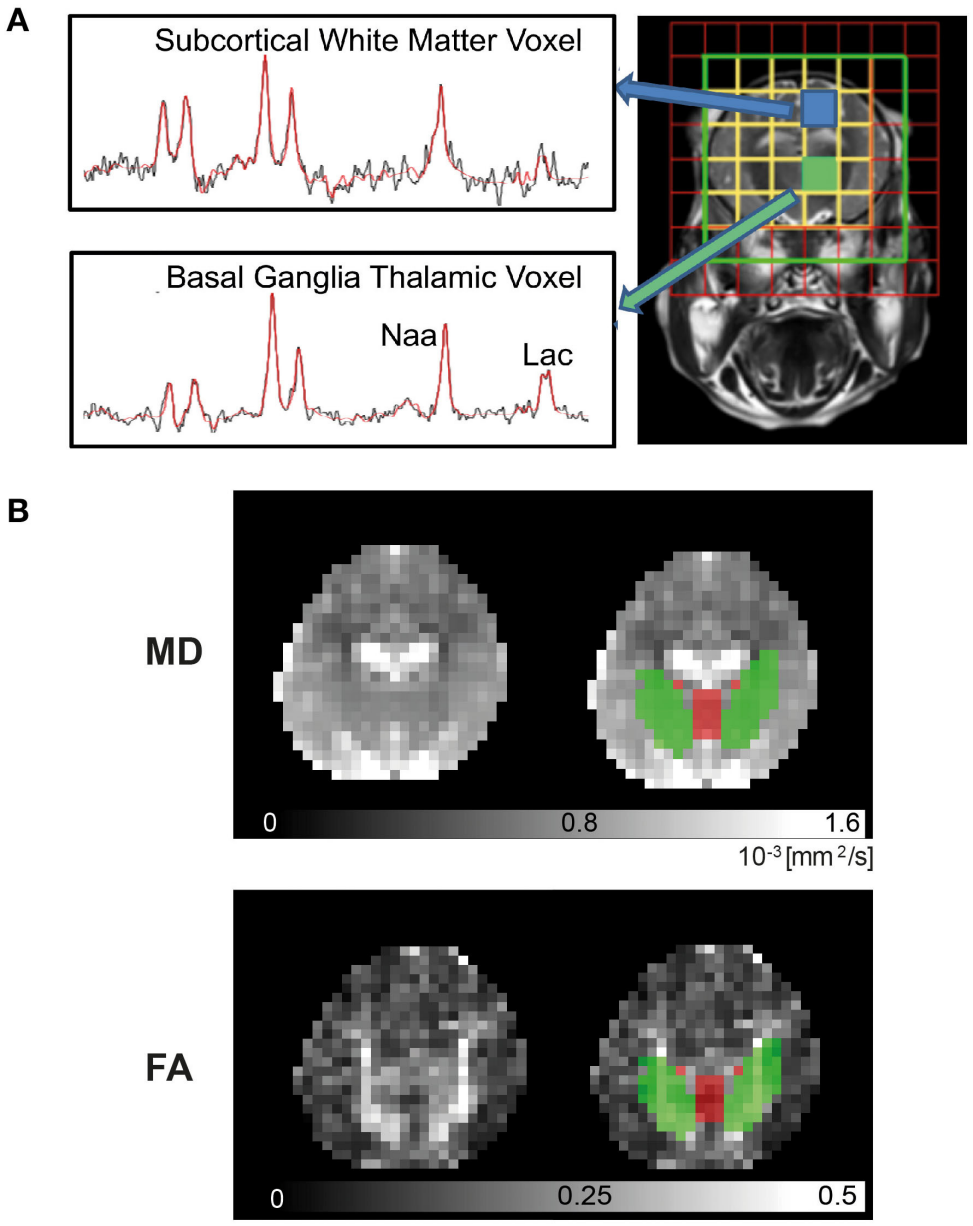
3T Magnetic Resonance Imaging. Piglets were scanned at 24 and 48 h in the Philips Achieva clinical magnet. ^1^H- Magnetic Resonance Spectroscopy (MRS) **(A)** was acquired using chemical shift imaging (CSI) with 8 × 8 matrix and 8 × 8 × 10 mm^3^ voxels, TR/TE was 2,000 ms/288 ms. The spectral width was 2 kHz with 2,048 points. Voxels selected were over the left thalamus [basal ganglia, thalamus (BGT)] and left subcortical white matter (WM) at the level of the centrum semiovale. Spectra were processed with Tarquin included in basis set, and the ratio of lactate (Lac)/N-acetylaspartate (NAA) was calculated from the amplitude of the fitted components (Lac+Thr/NAA+NAAG). For diffusion-weighted imaging (DWI) **(B)**, regional of interest (ROI) for mean diffusivity (MD) and fractional anisotropy (FA) in the deep gray matter (DGM) (red) and WM (green) were automatically identified by atlas label propagation. The internal capsule was selected to represent WM, and the caudate, putamen, globus pallidus, thalamus, and hypothalamus were used for DGM.

Piglets were transferred to the 3T MRI scanner at 24 and 48 h post insult. Imaging was performed with similar protocols as those used in NE babies on the same 3T scanner (8). ^1^H MRS was acquired with 8 × 8 matrix and 8 mm^3^ × 8 mm^3^ × 10 mm^3^ voxels with TR/TE 2,000 ms/288 ms. The spectral width was 2 kHz with 2,048 points. MRS data for the BGT (left thalamus) and WM voxels (left subcortical WM at the level of centrum semiovale level) were selected (Figure 2A) and processed using Tarquin with threonine included in the basis set. Lipids and macromolecules were excluded. The ratio of Lac/NAA was calculated from the amplitude of the fitted components (Lac+Thr/NAA+NAAG). Other metabolite peaks obtained include choline (Cho), and creatine (Cr) to give Lac/Cho, Lac/Cr, NAA/Cho, NAA/Cr, and Cho/Cr ratios.

Diffusion-weighted imaging (DWI) was acquired using a protocol similar to clinical studies (Figure 2B) (8). DWI was acquired with diffusion sensitizing gradient in 16 directions, with b-value of 750 s/mm^2^, echo planar imaging (EPI) readout: TR = 9,000 ms, TE = 61 ms, slice thickness = 2 mm, in-plane resolution 2.0 mm^2^ × 2.0 mm^2^, slice thickness = 2 mm, 20 slices. Postprocessing of the data was carried out using FSL brain imaging software library (8). Brain tissue was manually segmented using ITK-SNAP (18), and DWI volumes were corrected for eddy current-induced distortions with FSL-EDDY tool. Deep gray matter (DGM) and WM regions were identified automatically by atlas (19) labels propagation. First, high-resolution structural template scans were co-registered to each piglet structural scan that was resampled to isotropic voxel size. DWI data and structural scans were then co-registered, and a combination of transformations was used to propagate and down sample labels using nearest neighbor interpolation. Finally, basal ganglia nuclei and thalami were combined into a single DGM region, and MD and FA were calculated within DGM, internal capsule, and whole-brain masks.

### Histology

Brain histology and immunohistochemistry samples were prepared as previously described (11, 12). In brief, experiments were terminated 48 h post HI, and the piglets were euthanized with pentobarbital. Following this, piglets underwent cold phosphate buffered saline (PBS) cardiac perfusion and tissue fixation with 4% paraformaldehyde (PFA). The brain was dissected and stored in 2% PFA. Then, 5-mm coronal slices were made from the right hemisphere, embedded in paraffin, and cut into 8-μm sections. Two slices were selected for use for histology analysis: R0 at the level of the optic chiasm and R1 at the level of the hippocampus. These were dehydrated in xylene and rehydrated in graded ethanol solution (100–70%) prior to immunohistochemistry to stain cell death (TUNEL), microglia activation (Iba1), and apoptosis (CC3).

TUNEL was used to assess cell death. As previously described (12), slices were treated with 3% hydrogen peroxide followed by predigestion with protease K (Promega, Southampton, UK) and finally incubated in TUNEL solution for 2 h (Roche, Burgess Hill, UK). To visualize the biotin residues, slices were incubated in avidin-biotinylated horseradish peroxidase complex (ABC, Vector Laboratories) followed by diaminobenzidine/H_2_O_2_ (Sigma) with CoCl_2_ and NiCl_2_. A hematoxylin–eosin counterstain was applied, and slices were mounted on coverslips with dibutylphthalate polystyrene xylene (DPX).

For each piglet, eight regions of the brain were examined. In seven regions, TUNEL-positive nuclei were counted (Figure S1) from three fields in each of the R0 and R1 slices at 40× magnification. The hippocampus was present in the R1 section only. The counts were converted into cell counts per mm^2^.

To assess microglia activation, slides were prepared as previously described by Martinello et al. (14) and Ito et al. (20). Brain sections were pretreated in Ventana CC1 (950-124) and incubated in primary rabbit antibody anti-Iba1 polyclonal antibody (1:250) (WAKO 019-19741) for 4 h followed by incubation in secondary swine anti-rabbit immunoglobulin (DAKO E0343) for a further 1 h. Slices were mounted with Vectrashield + 4',6-diamidino-2-phenylindole (DAPI) aqueous mounting media. The Iba1-positive microglia cell bodies and branch density were calculated using a 0.049 mm × 0.049 mm square grid under 40× magnification. The number of cell bodies was counted within the grid (C), and the average number of branches crossing the three horizontal and vertical grid lines (B) was counted to give a microglial ramification index (B^2^/C).

For CC3 immunohistochemistry, brain sections were pretreated as for Iba1 staining, incubated in rabbit anti-CC3 (1:100) (Cell Signaling 9661L) for 32 min followed by swine anti-rabbit immunoglobulin for 44 min. Sections were mounted on Vectrashield + DAPI as described above. CC3 cells were counted at 20× magnification in three fields per brain region and converted to counts per mm^2^.

### Data and Statistical Analysis

Data analysis was carried out using SPSS Statistics 24 (IBM). The overall whole-brain TUNEL-positive cell counts, CC3-positive cell counts, and Iba1 ramification index were deduced from the sum of the average counts in eight regions of the brain (Figure S1).

The 24 and 48 h MRS data were collected for each of the BGT and WM regions and separately correlated with average whole-brain TUNEL, CC3, and Iba1 counts. In addition, the overall mean MRS FA and MD values were deduced from the 24 and 48 h scans and compared with the three immunohistochemistry markers.

The MRS, DWI, and histology count values were log_10_ transformed to normalize the distribution. The correlation was assessed using Pearson's rank coefficient, and scatter plots were created with GraphPad Prism v8 to illustrate the trend. *P*-values were calculated with two-tailed test to indicate statistical significance. As we compared multiple independent tests, the threshold for statistical significance was corrected to preserve a type 1 error rate (where *p* < 0.05 is significant) using Bonferonni correction. A *p* < 0.01 denotes statistical significance. Logistic regression modeling in infants with NE identified Lac/NAA of 0.39 as the optimal cutoff value for sensitivity and specificity to predict adverse neurodevelopmental outcomes at 18 months (8). Using this clinical Lac/NAA value, the mean log_10_ TUNEL, Iba1, and CC3 counts were deduced, and significance was compared using independent *t*-test.

## Results

Sixty-seven male large white piglets were recruited from four neuroprotection studies including 11 (16.4%) from Robertson et al. (11); 28 (41.8%) from Robertson et al. (17), and Lingam et al. (13); and 16 (23.9%) from Martinello et al. (14). Twelve piglets were included from an unpublished study. Twenty-seven piglets were excluded as no 3T MRS data were available. There was a larger proportion of piglets excluded from study 1 (*n* = 17/27, 60.7%) as scans at 3T were introduced later in this study. MRS data at both the 24 and 48 h MRI scans were complete for 54 piglets (80.6%). The remaining 13 piglets had either 24 h scan (*n* = 8) or 48 h scan (*n* = 5) available. Reasons for incomplete data include piglet death prior to the 48 h scan (*n* = 4), 3T scanner not available due to technical issues (*n* = 3), or issues with the spectral fit processing (*n* = 6).

Figure 1 summarizes the treatment regimens across the four studies. In total, 59 of 67 (88.1%) piglets were subjected to cerebral injury. The remaining eight animals were either naive (*n* = 3, 4.5%) or LPS inflammation sensitized without cerebral injury (*n* = 5, 7.5%). Cerebral injury included: HI (39/67, 58.2%), LPS inflammation sensitized hypoxic injury (8/67, 11.9%), and LPS inflammation sensitized HI injury (12/67, 17.9%).

Neuroprotective interventions also varied with 45 of 67 (67.2%) piglets receiving HT for either 12 h (34/67, 50.7%) or 24 h (*n* = 11/67, 16.4%). Melatonin was given to 11 (16.4%) animals, and magnesium was given to eight (11.9%) piglets.

### Lactate/N-Acetylaspartate to Terminal Deoxynucleotidyl Transferase dUTP Nick End Labeling

**Figure 3:**
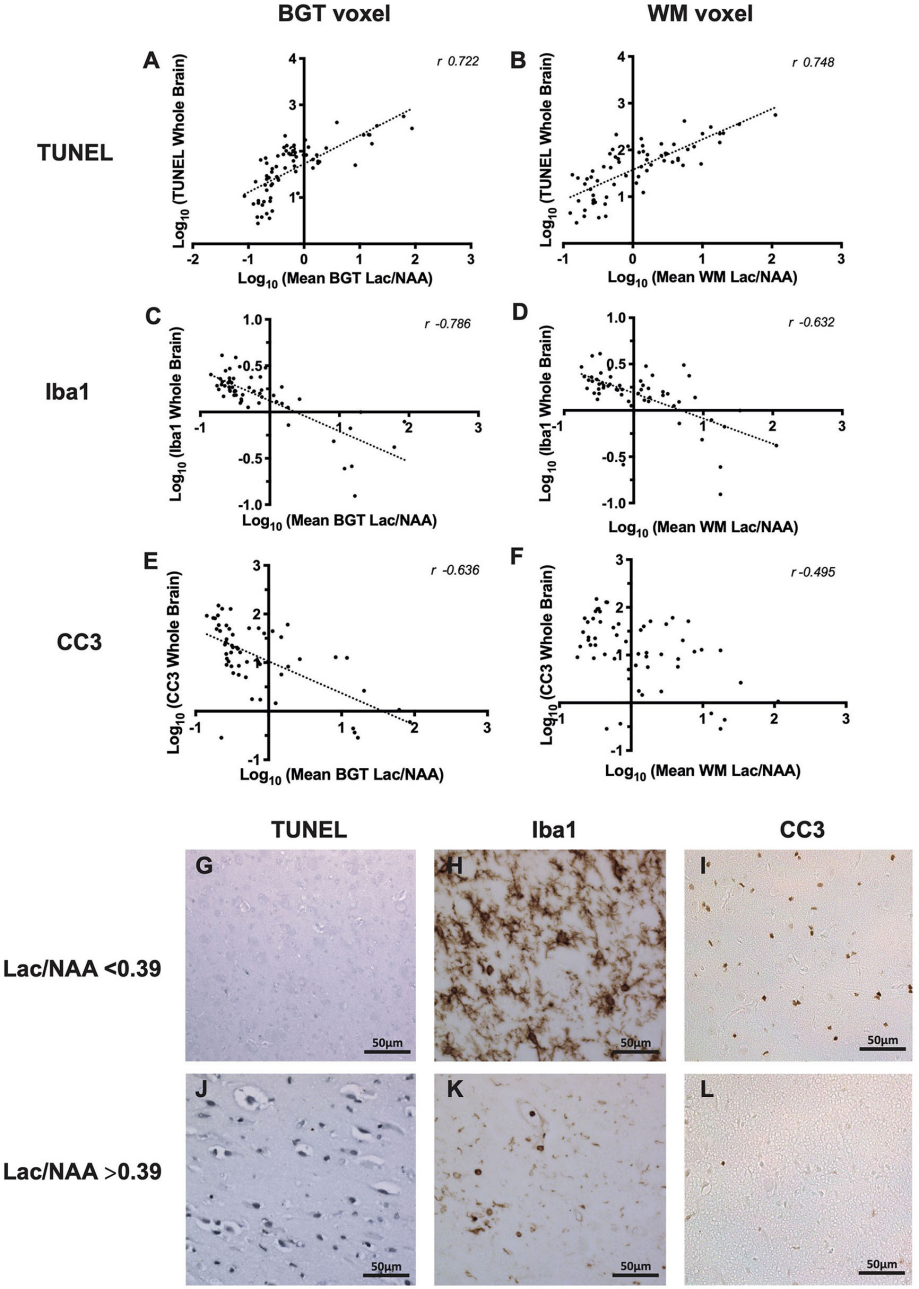
The relationship between thalamic [basal ganglia, thalamus (BGT)] and white matter (WM) ^1^H magnetic resonance spectroscopy (MRS) lactate (Lac)/N-acetylaspartate (NAA) and whole-brain cell death [terminal deoxynucleotidyl transferase dUTP nick end labeling (TUNEL)] **(A,B)**, microglia activation [ionized calcium-binding adapter molecule 1 (Iba1) ramification index] **(C,D)**, and cleaved caspase 3 (CC3) **(E,F)**. All data in the scatterplots were log_10_ transformed, and the Pearson's correlation coefficients (*r*-values) are illustrated. All values *p* < 0.001. Representative micrographs showing TUNEL, Iba1, and CC3 immunohistochemistry stains in piglets with Lac/NAA <0.39 **(G–I)** and Lac/NAA ≥0.39 **(J–L)** are shown.

Mean Lac/NAA and TUNEL-positive cell counts of the whole brain correlated in the BGT (*r* = 0.722, *p* < 0.001) and WM voxel (*r* = 0.784, *p* < 0.001) (Figures 3A,B). The positive correlation was present at both 24 and 48 h (Table 1).

**Table 1:** Pearson's correlation coefficient comparing magnetic resonance spectroscopy (MRS) lactate (Lac)/N-acetylaspartate (NAA), diffusion-weighted imaging (DWI) mean diffusivity and fractional anisotropy at 24 and 48 h and the mean of the two time points with whole-brain terminal deoxynucleotidyl transferase dUTP nick end labeling (TUNEL)-positive count.

	**MRS Lac/NAA**		**DWI Mean Diffusivity**		**DWI Fractional Anisotropy**	
	***r***	***p*-value**	***r***	***p*-value**	***r***	***p*-value**
24 h BGT region	0.709	<0.001	−0.488	<0.001	0.424	<0.001
48 h BGT region	0.661	<0.001	−0.635	<0.001	0.240	0.065
**Mean BGT region**	0.722	<0.001	−0.615	<0.001	0.523	<0.001
24 h WM region	0.669	<0.001	−0.511	<0.001	0.452	<0.001
48 h WM region	0.729	<0.001	−0.633	<0.001	0.495	<0.001
**Mean WM region**	0.748	<0.001	−0.635	<0.001	0.342	0.005

*All values were log_10_ transformed prior to correlation analysis. Correlation is significant if p < 0.01 (corrected with Bonferroni correction where p < 0.05 is considered significant). BGT, basal ganglia and thalamus; WM, white matter*.

Lac/NAA ≥0.39 was associated with significantly higher TUNEL-positive cells in the whole brain across both voxels and both the 24 and 48 h scans (Table 2, Figures 3G,J) (*p* < 0.001) [mean BGT Lac/NAA ≥0.39, mean TUNEL count = 103 cells/mm^2^ (SD 2.12); mean BGT Lac/NAA <0.39, mean TUNEL count = 15.2 cells/mm^2^ (SD 2.56); *p* < 0.001].

**Table 2:** Histology cell counts using a clinical threshold for lactate (Lac)/N-acetylaspartate (NAA) of 0.39.

	**Whole-Brain TUNEL Count**		***p*-value**	**Whole-Brain Iba1 Ramification Index**		***p*-value**	**Whole-Brain CC3**		***p*-value**
	**Lac/NAA <0.39**	**Lac/NAA ≥0.39**		**Lac/NAA <0.39**	**Lac/NAA ≥0.39**		**Lac/NAA <0.39**	**Lac/NAA ≥0.39**	
BGT voxel at 24 h	18.2 (SD 2.96)	109 (SD 2.03)	<0.001	1.96 (SD 1.30)	0.88 (SD 2.08)	<0.001	25.8 (SD 4.94)	4.89 (SD 4.94)	<0.001
BGT voxel at 48 h	15.1 (SD 2.84)	83.4 (SD 2.42)	<0.001	2.05 (SD 1.29)	1.05 (SD 2.05)	<0.001	27.9 (SD 3.75)	6.78 (SD 4.83)	0.001
**Mean BGT voxel**	15.2 (SD 2.56)	103 (SD 2.12)	<0.001	1.94 (SD 1.29)	0.96 (SD 2.13)	<0.001	25.0 (SD 3.47)	5.82 (SD 5.19)	0.001
WM voxel at 24 h	15.9 (SD 3.26)	84.7 (SD 2.27)	<0.001	1.87 (SD 1.61)	1.11 (SD 1.96)	0.004	24.2 (SD 4.76)	7.71 (SD 4.39)	0.008
WM voxel at 48 h	12.2 (SD 2.61)	58.3 (SD 3.15)	<0.001	2.09 (SD 1.36)	1.29 (SD 1.96)	0.003	27.4 (SD 2.23)	10.7 (SD 5.90)	0.076
**Mean WM voxel**	11.3 (SD 2.27)	71.6 (SD 2.75)	<0.001	2.04 (SD 1.29)	1.17 (SD 1.99)	0.003	33.0 (SD 2.37)	8.08 (SD 5.41)	0.001

*Values shown are the geometric mean and standard deviation (SD). Using this threshold, we showed significant differences in cell counts for cell death [terminal deoxynucleotidyl transferase dUTP nick end labeling (TUNEL)] and microglial activation [ionized calcium-binding adapter molecule 1 (Iba1) ramification index] in the piglets. Correlation is significant if p < 0.01 (corrected with Bonferroni correction where p < 0.05 is considered significant). BGT, basal ganglia and thalamus; WM, white matter*.

### Lactate/N-Acetylaspartate to Ionized Calcium-Binding Adapter Molecule 1

Iba1 ramification index was used to assess microglial activation. Activated microglia become ameboid with fewer processes, represented by a lower ramification index. There was a strong negative correlation between mean Lac/NAA and whole-brain Iba1 ramification index (Figures 3C,D). The negative correlation was strongest with the mean BGT voxel (*r* = −0.786, *p* < 0.001) but also present in the mean WM voxel (*r* = −0.632, *p* < 0.001).

Using a Lac/NAA threshold of 0.39, we noted significant differences in the Iba1 ramification index between piglets at all time points and voxels (*p* < 0.001) (Table 2, Figures 3H,K). Lac/NAA ≥0.39 was associated with lower Iba1 ramification (mean BGT Lac/NAA ≥0.39, Iba1 ramification index 0.96 vs. 1.94 with Lac/NAA <0.39) (*p* < 0.001).

### Lactate/N-Acetylaspartate to Cleaved Caspase 3

The correlation between Lac/NAA and CC3 was negative in the BGT voxel (*r* = −0.636; *p* < 0.001) but was weaker in the WM voxel (*r* = −0.495; *p* < 0.001) (Figures 3E,F).

Lac/NAA ≥0.39 was associated with lower CC3 counts at 24 h (CC3 count 4.89 vs. 25.8 cells/mm^2^, *p* < 0.001) and 48 h scans (CC3 count 6.78 vs. 27.9 cells/mm^2^, *p* < 0.01) in the BGT voxel. The CC3 count was also significantly lower with Lac/NAA ≥0.39 at 24 h in the WM voxel (CC3 count 7.71 vs. 25.0, *p* < 0.01) (Table 2, Figures 3I,L).

### Magnetic Resonance Spectroscopy Metabolite Peak Ratios to Terminal Deoxynucleotidyl Transferase dUTP Nick End Labeling

Pearson's correlation coefficients were deduced to compare other proton MRS metabolite ratios with whole-brain TUNEL count (Table 3). We observed strong positive correlations between the total whole-brain TUNEL count and mean BGT Lac/Cho (*r* = 0.765, *p* < 0.001) and BGT Lac/Cr (*r* = 0.765, *p* < 0.001). There was a lesser correlation in the corresponding WM voxels (WM Lac/Cho *r* = 0.701, *p* < 0.001; Lac/Cr 0.671, *p* < 0.001). There was a weak correlation between BGT NAA/Cho (*r* = −0.530, *p* < 0.01) and BGT NAA/Cr (*r* = −0.565, *p* < 0.001) with TUNEL. There was no correlation between TUNEL and Cho/Cr (BGT voxel, *r* = 0.019, *p* = 0.88; WM voxel, *r* = −0.051, *p* = 0.68).

**Table 3:** Pearson's correlation coefficient comparing magnetic resonance spectroscopy (MRS) metabolite ratios with whole-brain terminal deoxynucleotidyl transferase dUTP nick end labeling.

		***r***	***p*-value**
Mean Lac/NAA	BGT	0.722	<0.001
	WM	0.748	<0.001
Mean Lac/Cho	BGT	0.765	<0.001
	WM	0.701	<0.001
Mean Lac/Cr	BGT	0.766	<0.001
	WM	0.671	<0.001
Mean NAA/Cho	BGT	−0.530	<0.001
	WM	−0.565	<0.001
Mean NAA/Cr	BGT	−0.565	<0.001
	WM	−0.624	<0.001
Mean Cho/Cr	BGT	0.019	0.877
	WM	−0.051	0.684

*Correlation is significant if p < 0.01 (corrected with Bonferroni correction where p < 0.05 is considered significant). BGT, basal ganglia and thalamus; Cho, choline; Cr, creatine; Lac, lactate; NAA, N-acetylaspartate; WM, white matter*.

When compared with other metabolic ratios Lac/NAA was most consistent in yielding similarly strong correlation co-efficient values in the BGT and WM voxels.

### Diffusion-Weighted Imaging to Terminal Deoxynucleotidyl Transferase dUTP Nick End Labeling

**Figure 4:**
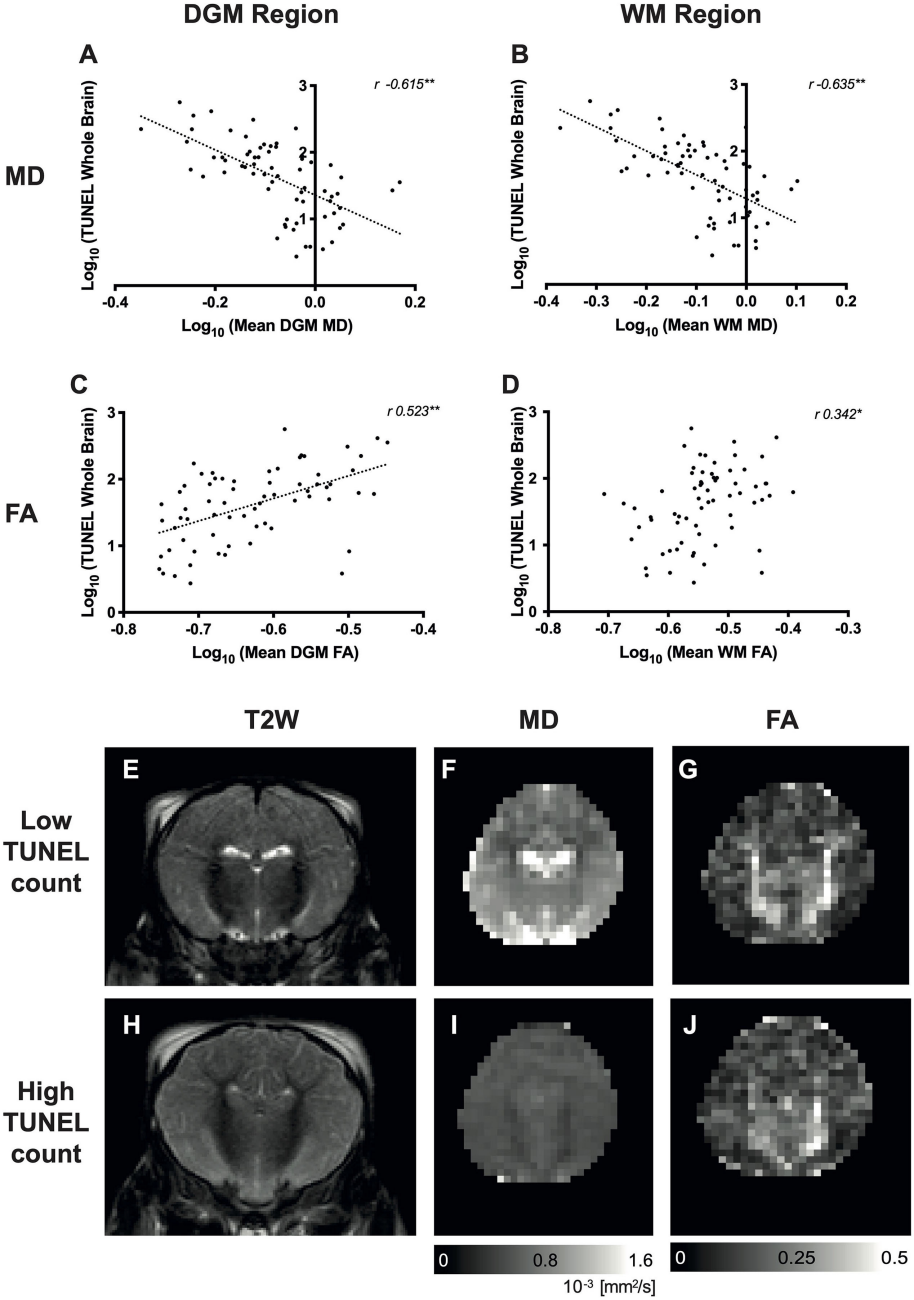
Relationship between diffusion-weighted imaging (DWI) and whole-brain cell death [terminal deoxynucleotidyl transferase dUTP nick end labeling (TUNEL)-positive cells]. Scatterplots showing the correlation between whole-brain TUNEL and average mean diffusivity (MD) **(A,B)** and fractional anisotropy (FA) **(C,D)** localized in the deep gray matter (DGM), white matter (WM) regions. Data were log_10_ transformed, and the Pearson's correlation coefficients (*r*-values) are illustrated. *p < 0.01, **p < 0.001. Representative T2-weighted images (T2W), MD and FA maps are shown for a piglet with low TUNEL-positive counts (**E–G**, respectively) and high TUNEL-positive counts **(H–J)**.

The correlation between DWI MD and TUNEL was negative (mean DGM MD to TUNEL *r* = 0.615, *p* < 0.001; mean WM MD to TUNEL *r* = −0.635, *p* < 0.001) as illustrated in Figures 4A,B; however, the correlation was not as a strong as between Lac/NAA and TUNEL-positive cells (Table 3). The correlation between FA and TUNEL was weak at 24 and 48 h (mean DGM *r* = 0.523, *p* < 0.001; mean WM *r* = 0.342, *p* < 0.01) (Figures 4C,D; Table 1). Representative T2-weighted images (T2W), MD and FA maps are shown in Figures 4E–J.

## Discussion

^1^H MRS Lac/NAA peak area ratio correlated with overall TUNEL-positive cell death and microglial activation in a piglet model of term perinatal brain injury. Compared to other MRS metabolite ratios and DWI, Lac/NAA demonstrated the best correlation to TUNEL-positive cell death at 24 and 48 h. This concurs with studies in cooled infants with NE where BGT Lac/NAA peak area ratio has a high level of accuracy for outcome prediction compared to other MR methods (8, 9). Using the same Lac/NAA peak area ratio threshold of 0.39 identified in clinical settings (8), we demonstrate significant differences in TUNEL-positive cells and microglial activation. These data support the translational relevance of Lac/NAA in preclinical and clinical neuroprotection studies. In our piglet model, we observed strong correlations between BGT Lac/Cr and Lac/Cho and whole-brain TUNEL; however, these were not consistent in the WM MRS voxel (21–23).

The combined increased lactate and reduced NAA on MRS (translating to a high Lac/NAA peak area ratio) suggest brain mitochondrial impairment and impaired oxidative metabolism during “secondary energy failure.” It is possible that this ongoing injury may be amenable to late therapies and thus is an important measure to direct therapies. Woo et al. (24) previously demonstrated a correlation between MRS lactate and NAA with TUNEL-positive cells in a rat model. In this normothermic, middle cerebral artery occlusion model, Lac/Cr increased immediately after reperfusion, whereas NAA/Cr decreased 9 h after injury. There was a strong correlation between Lac/Cr and NAA/Cr at 24 h, and both these metabolite ratios correlated with TUNEL in the basal ganglia. Interestingly, Lac/Cr did not correlate with brain infarct volume at 4 weeks, which they argued was due to using a single voxel that may not reflect whole-brain injury. In our study, we show that Lac/NAA in both the deep gray matter and WM correlates with brain cell death across eight brain regions. Our data concur with those from a lamb model of birth asphyxia involving umbilical cord occlusion; there was a strong correlation between TUNEL in the thalamus and deep gray matter and Lac/NAA at 72 h (25). Our study goes further as we investigated relationships between both TUNEL cell death and neuroinflammation in a variety of perinatal injuries and neuroprotective interventions.

Lac/NAA peak ratio in our piglet studies represents more precisely Lac+threonine/total NAA. Mitra et al. (8) describes the optimization of metabolite fitting with the inclusion of threonine in the spectra. Threonine is an amino acid present in the brain, and the resonance of its methyl groups overlaps with that of lactate on the spectra at 1.3 ppm. The addition of threonine in the spectra fit is important in the accurate quantification of lactate (26).

The source and exact mechanism of raised cerebral lactate remain unknown. Both neurons and astrocytes produce lactate in hypoxic conditions *in vitro* (27). Lactate is a product of anaerobic respiration, produced by lactate dehydrogenase from pyruvate regenerating NAD+ for glycolysis. In traumatic brain injury (TBI) models (28, 29), it is thought that lactate reflects the redox state of NADH/NAD+. NADH is a product of glycolysis and the Krebs cycle, which is oxidized in the electron transport chain of the mitochondria to build a proton gradient for ATP production. Following HI, mitochondrial failure and disruption of the electron transport chain lead to the buildup of NADH and reduction in ATP levels. As a result, the equilibrium may shift toward lactate and NAD+ production to rebalance the intracellular NADH/NAD+ ratio. Pellerin and Magistretti (30) proposed the astrocyte to neuron lactate shuttle (ANLS) model whereby lactate, generated by astrocytes through their high glycolytic metabolism capacity, is shuttled to neurons *via* monocarboxylate transporters (MCTs) and metabolized in neurons to pyruvate as an alternative fuel to glucose. In a rat study of severe traumatic brain injury, uncoupling of the ANLS was associated with disruption in the neuronal uptake of lactate (31), thereby contributing to the rise in lactate. This suggests that where neurons are too damaged to utilize the lactate produced by astrocytes, i.e., uncoupling of neuronal and glial metabolism, high extracellular levels of lactate would accumulate, explaining the association between high lactate and poor outcome (29). Other proposed mechanisms of elevated lactate include increased phagocytes (which exhibit increased anaerobic activity), gliosis, altered Na^+^/H^+^ buffer system (32, 33), and influx of lactate from other injured tissues (34).

There is increasing interest in the role of lactate as a neuroprotective agent. In adults with traumatic brain injury, administration of ^13^C-labeled lactate *via* the microdialysis catheter and simultaneous collection of the microdialysates, with ^13^C NMR analysis, revealed ^13^C labeling in glutamine, consistent with lactate metabolism in the TCA cycle (31, 35). Interestingly, Roumes et al. (36) recently demonstrated the neuroprotective effect of exogenous lactate administration in a neonatal HI rat model. Rice-Vannucci P7 rats that received intraperitoneal injection of lactate following unilateral carotid ligation exhibited a significant reduction in the volume of high-signal intensity brain lesions on DWI and reduced severity of cytotoxic edema as demonstrated by higher apparent diffusion coefficient (ADC) values compared to animals that received 0.9% sodium chloride. Interestingly, rats with HI injury that received three daily injections of lactate performed as well as sham animals without brain injury in sensorimotor and memory neurobehavioral tests. It was proposed that the uptake of lactate by astrocytes, transported *via* the ANLS to neurons, provided an alternative source of energy, thereby sparing the limited glucose for use in the pentose phosphate pathway for glutathione production, a potent reactive oxygen species (ROS) scavenger. Lactate dehydrogenase inhibition using oxamate negated the neuroprotective effects of lactate, demonstrating a role of lactate in neuronal energy metabolism and a link to its neuroprotective properties. A reduction in ROS production was also observed in animals treated with lactate, which was lost when co-administrated with oxamate. We did not perform co-localization immunohistochemistry in all our studies; however, in study 1 (11), co-labeling with TUNEL and glial fibrillary acidic protein (GFAP) in the sensorimotor cortex demonstrated that the majority of TUNEL-positive cells did not co-localize with GFAP, suggesting that the dying cells were not astrocytes. The increased brain lactate that we observe in the most damaged brains after HI may thus reflect astrocytic activity to provide lactate for neuronal needs and an uncoupling of the ANLS.

NAA is a metabolite produced by aspartate N-acetyl transferase and found in neurons. NAA is transported from neurons to oligodendrocytes where it is metabolized into aspartate and acetate and used for energy production and myelin synthesis, respectively (37). NAA has been described as a surrogate marker of neuronal density, integrity, and metabolic activity (38) and of neuronal viability (39). Reduction in absolute concentration of NAA (9) and relative peak ratio of NAA (40), measured with MRS, alone predicts poor neurodevelopmental outcomes in babies with NE (23, 40). Lally et al. (9) reported the predictive accuracy of absolute [NAA] for 2-year cognitive, language, and motor outcomes (AUC 0.99), although Lac/NAA peak area ratio was also highly predictive (AUC 0.94). In our current study, the correlation between NAA/Cho and NAA/Cr with TUNEL was negative but weak compared to ratios that included lactate; this is supported by clinical studies showing NAA/Cho and NAA/Cr are less predictive in neurodevelopmental outcomes compared to Lac/NAA (21–23), although this may be due to inconsistencies in Cho and Cr measurement (39, 40).

We noted a negative correlation between DWI MD and TUNEL-positive cells in the brain. In babies with NE, MD pseudonormalizes at around 7 days in non-cooled infants or 10 days in infants who received HT (41). Our findings in our preclinical model and in babies with NE concur with the experience in a comparative stroke study, where MRS has been shown to better predict outcomes compared with MD. In this study, recovery to normal values of ADC occurred despite the subsequent infarction of tissue, whereas NAA levels continued to show a decline in the same area, thus reflecting tissue injury more accurately (42). Nevertheless, in clinical studies of NE, lower DWI-MD is associated with adverse neurodevelopmental outcomes (41, 43). FA may have more utility in the prediction of outcome as pseudonormalization does not occur; however, we showed poor correlation with TUNEL-positive cell death, which concurs with results from the TOBY Xe neuroprotection study in babies, where FA added little extra to Lac/NAA in accurately predicting neurodevelopmental outcome (10).

In our experience, although highest levels of Lac are seen in the first few days after birth, brain Lac persists for months in babies with adverse outcome after NE; this persisting brain Lac is associated with abnormal MRI and brain alkalosis (32, 44). In our study, piglets were scanned at 24–48 h, which is earlier than in clinical studies of NE [mean age 8.4 days (9) and 6 days (40), respectively]. Therefore, in our preclinical studies, the Lac component in Lac/NAA may have more influence on prediction than NAA in the early post HI period. Wu et al. (34) demonstrated significantly higher cerebral Lac levels early after HI in infants with moderate to severe encephalopathy, which progressively reduced over several days. It is likely that, in the early phase after injury, Lac levels drive the predictive accuracy, whereas in the later phases, the reduction in NAA drives the predictive accuracy (NAA reduction occurs more gradually than the acute Lac rise) (32). A further advantage of combining Lac/NAA peak area ratio is that they depend on both metabolite T2 relaxation times and concentrations, both of which are pathologically modulated, and hence injury severity prediction is improved (39).

Microglia are one of the first inflammatory cells to be activated following HI (45). We used Iba1 ramification index to quantify the morphology of the microglia. At rest, microglia are highly ramified cells with multiple processes sensing the environment (46). Once activated, microglia exhibit a larger body and fewer processes, measured histologically by a reduction in the ramification index. We demonstrated a strong correlation between the degree of microglial activation (Iba1 ramification index) and Lac/NAA, particularly in the BGT, supporting Lac/NAA as a biomarker for neuroinflammation. The role of microglia in secondary energy failure includes cytokine release, metalloproteinase production, breakdown of the blood–brain barrier, leukocyte infiltration, and ultimately further brain injury (46). We have previously shown that piglets with LPS sensitization 4 h prior to hypoxia resulted in an increase in mortality and overall brain cell death (TUNEL-positive cells), particularly in the internal capsule, periventricular WM, and sensorimotor cortex (14). In addition, microglial activation can persist for many years after insult, and it has been proposed that this pathological activation leads to altered neurogenesis and synaptogenesis (47) and persisting brain Lac.

We observed a poor correlation between Lac/NAA and CC3, and this relationship was unexpectedly negative. This finding concurs with our previous neuroprotection studies where we see little relation between CC3 and other markers of injury severity, particularly TUNEL-positive cells (11). The scatterplots indicate several outliers and a non-linear relationship. The reason for this disconnect between CC3 and brain injury markers is likely to be related to several factors including: (i) cell death occurring by processes independent of caspase, such as necrosis, necroptosis, and autophagy; (ii) sexually dimorphic cell death pathways; as our piglets included only males (in whom cell death occurs through caspase-independent routes such as poly(ADP-ribose) polymerase (PARP)-dependent cell death pathways), CC3 will be a poor marker of cell death (48); (iii) LPS can cause an increase in CC3 without resulting in cell death (49, 50), reflecting the alternative non-apoptotic functions of CC3 (51); (iv) most piglets in this study were treated with HT, which inhibits caspase 3 activation (52).

There are limitations to this study. These data were retrospective and obtained from different studies with differing insults and neuroprotective interventions. However, this is also a strength of the study as the strong correlation of Lac/NAA to TUNEL-positive cells supports the validity of this biomarker across perinatal brain injury which is frequently multifactorial and heterogeneous in nature. In this study, some animals were cooled for 12 h, rather than 24 h (17). This was justified as we develop the model to reflect the clinical situation where cooling is partially effective as in babies with NE; furthermore, the efficacy of HT over 12 h cooling has previously been demonstrated (53). Studies lasted only 48 h after HI, and as mentioned, this early phase will reflect higher Lac levels. In addition, according to the resolution of the DWI derived parameter maps (MD and FA), partial volume effects that could bias our result could not be excluded.

In conclusion, we describe a strong correlation between MRS Lac/NAA and TUNEL-positive cells and microglial activation across WM and gray matter in male piglets after a range of perinatal insults and neuroprotective interventions. These preclinical data concur with clinical studies that have demonstrated the utility of BGT Lac/NAA as a surrogate marker that best predicts outcome in NE and can be used to expedite early-phase clinical neuroprotection trials in NE.

## Data Availability Statement

The datasets generated for this study are available on request to the corresponding author.

## Ethics Statement

The animal study was reviewed and approved by UCL Ethics Committee.

## Author Contributions

RP organized and analyzed the data and drafted the manuscript with the help of KM. KM, AA-B, CM, IL, and TM undertook the experiments. CM undertook microscopy and cell quantification and organized the histology results. MS and AB scanned the piglets and collected MRS and DWI data. XG assisted with MR physics aspects of the study and reviewed the manuscript. NR obtained funding for the studies, designed the studies, and reviewed the manuscript. All authors reviewed the manuscript and approved the final version as submitted and agreed to be accountable for all aspects of the work.

## Conflict of Interest

The authors declare that the research was conducted in the absence of any commercial or financial relationships that could be construed as a potential conflict of interest.

## Abbreviations

3T: 3-Tesla
ADC: apparent diffusion coefficient
ANLS: astrocyte neuron lactate shuttle
AUC: area under the curve
BGT, basal ganglia: thalamus
CC3: cleaved caspase 3
Cho: choline
Cr: creatine
DGM: Deep gray matter
DWI: diffusion-weighted imaging
FA: fractional anisotropy
HI: hypoxia–ischemia
HT: therapeutic hypothermia
Iba1: ionized calcium-binding adapter molecule 1
Lac/NAA: lactate/N-acetylaspartate
Lac: lactate
LPS: lipopolysaccharide
MD: mean diffusivity
MRS: magnetic resonance spectroscopy
NAA: N-acetylaspartate
NAAG: N-acetylaspartylglutamate
NAD: nicotinamide adenine dinucleotide
NE: neonatal encephalopathy
RCT: randomized controlled trial
ROI: region of interest
T2W: T2 weighted
Thr: threonine
TUNEL: terminal deoxynucleotidyl transferase dUTP nick end labeling
WM: white matter.
