# Supplementary figures and images for: Proton Magnetic Resonance Spectroscopy Lactate/N-Acetylaspartate Within 48 h Predicts Cell Death Following Varied Neuroprotective Interventions in a Piglet Model of Hypoxia–Ischemia With and Without Inflammation-Sensitization
Source: Front Neurol. 2020 Sep 4;11:883. doi: 10.3389/fneur.2020.00883 (PMC7500093; doi:10.3389/fneur.2020.00883)

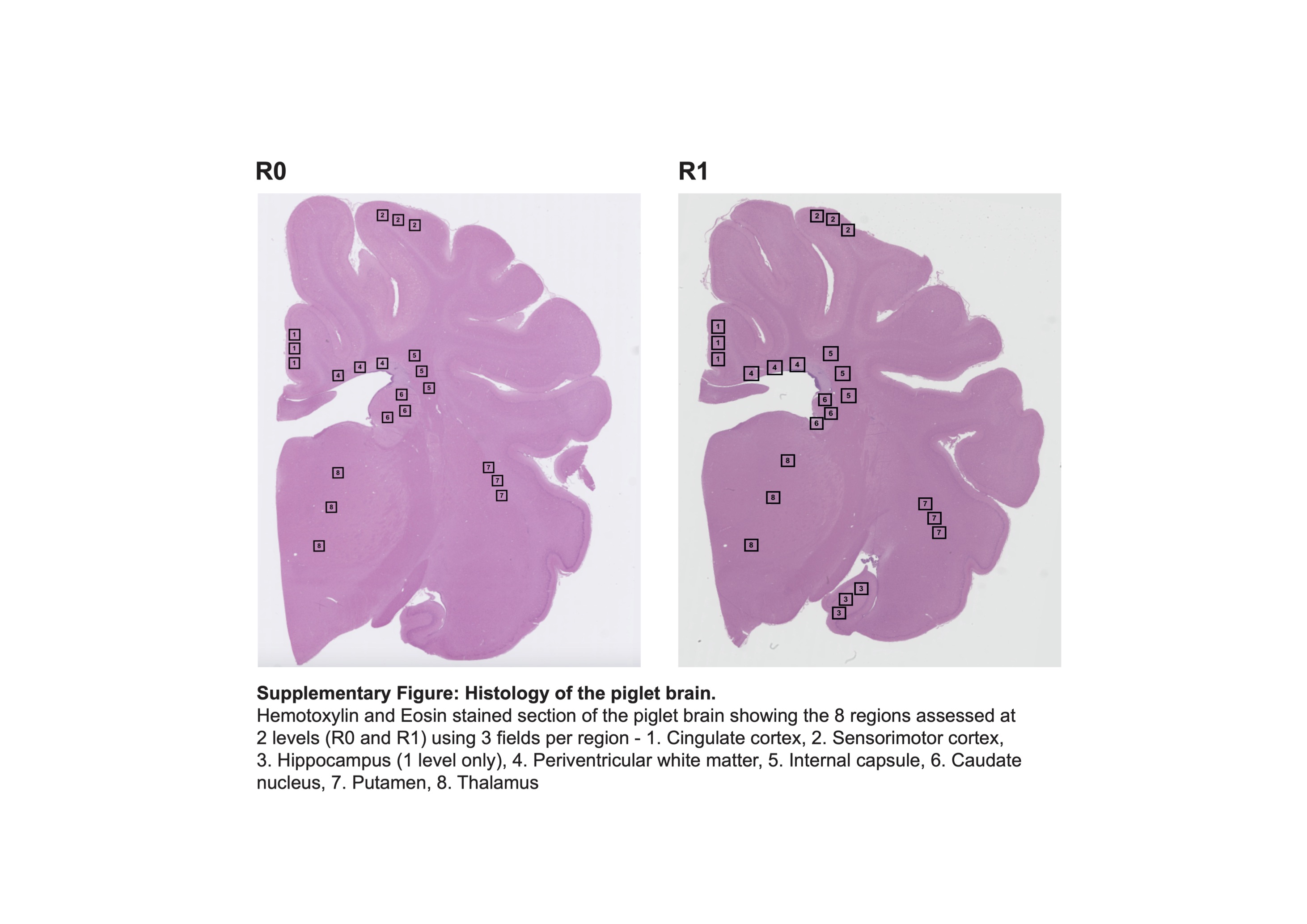

Supplement: Supplementary file 1 [file Image_1.jpg]
